# Associations between time spent in sedentary behaviors and metabolic syndrome risk in physically active and inactive European older adults

**DOI:** 10.1016/j.jnha.2025.100544

**Published:** 2025-03-23

**Authors:** Andreas Nilsson, Hadil Limem, Aurelia Santoro, Laura Smeldy Jurado-Medina, Agnes A.M. Berendsen, Lisette C.P.G.M. de Groot, Joanna Kaluza, Olga Januszko, Amy Jennings, Susan Fairweather-Tait, Claudio Franceschi, Fawzi Kadi

**Affiliations:** a School of Health Sciences, Örebro University, Sweden; b UFR STAPS, University of Paris Nanterre, France; c Department of Medical and Surgical Sciences, University of Bologna, Italy; d Interdepartmental Centre”Alma Mater Research Institute on Global Challenges and Climate Change (Alma Climate)”, University of Bologna, Italy; e Division of Human Nutrition, Wageningen University & Research, The Netherlands; f Department of Human Nutrition, Warsaw University of Life Sciences (WULS-SGGW), Poland; g Norwich Medical School, University of East Anglia, United Kingdom

**Keywords:** Aging, Physical activity, Metabolic syndrome, Sedentary time, Healthy diet

## Abstract

**Objectives:**

To determine how clustered metabolic risk based on a validated continuous metabolic syndrome risk score is associated with objectively assessed time in sedentary behaviors (SB) in physically active and inactive older adults, while considering adherence to healthy eating habits.

**Design:**

Cross-sectional study

**Setting and participants:**

The study comprises 871 community-dwelling older adults (age 65–79 years) recruited from four European countries.

**Measurements:**

Daily times spent in SB and physical activity (PA) were assessed by accelerometers (Actigraph GT3X) for a week. Waist circumference, triglycerides, HDL-cholesterol, mean arterial blood pressure, fasting glucose and insulin were determined, and a continuous metabolic syndrome risk score (cMSy) was generated using principal component analysis. Healthy eating habits were assessed by food record. General linear models stratified by adherence to PA guidelines (active/inactive) were used to examine differences in cMSy across tertiles of time in SB (Low, Medium, High) with adjustment for covariates, including healthy eating habits.

**Results:**

A significantly (*p* < 0.05) lower cMSy was observed among older adults in the low SB tertile compared to medium and high SB tertiles, with no difference between the latter tertiles. The favorable effect of low amounts of SB on cMSy was indicated in both active and inactive groups, and regardless of healthy eating habits. Further, being active was related to a more favorable cMSy across all SB tertiles.

**Conclusion:**

Low amounts of time spent in SB are related to a lower metabolic syndrome risk regardless of adherence to PA guidelines and healthy eating habits in older adults, supporting guidelines targeting limited amounts of SB alongside engagement in moderate-to-vigorous PA for promotion of metabolic health.

## Graphical abstract

**Figure:**
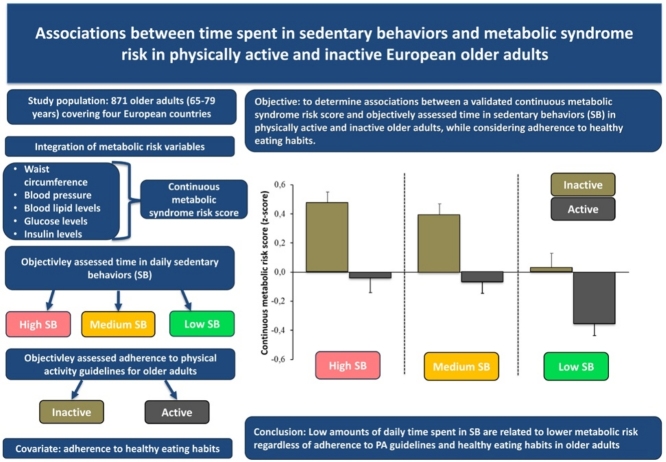

## 1 Introduction

A cluster of metabolic abnormalities, including insulin resistance, abdominal obesity, dyslipidemia, and hypertension, collectively recognized as the metabolic syndrome (MetS) [1], exacerbates the risk of developing cardiovascular diseases and type 2 diabetes [1,2]. As the prevalence of MetS increases by advancing age regardless of sex and ethnicity [3], older adults are a target for health-enhancing interventions aiming to mitigate the age-related progression of metabolic disorders. In this respect, physical activity (PA) represents an important lifestyle factor with potential to readily impact on cardiometabolic health [4]. Current global guidelines for promotion of metabolic health in older adults highlight the importance of engagement in ≥150 weekly minutes of moderate-to-vigorous PA (MVPA), while simultaneously limiting time spent in sedentary behaviors (SB) [4], the latter defined as any waking behavior with an energy expenditure ≤ 1.5 metabolic equivalents (METs), while in sitting, reclining or lying posture [5]. Although a quantified threshold to determine excessive amounts of SB for healthy aging is currently lacking, previous studies based on objective assessment of SB reveal that older adults spend most of the waking day (60–80%) in sedentary pursuits [[6], [7], [8]]. Moreover, a recent report shows that the proportion of adults with insufficient amounts of weekly MVPA (<150 min) increases after the age of 60 years [9], which together with prolonged times spent in SB exposes the older adult population to an increased cardiometabolic risk and related morbidities. In this respect, excessive amounts of time in SB have previously been linked to increased disease and mortality risk [[10], [11], [12], [13], [14], [15]]. However, to what extent time in SB acts as a risk factor for morbidity and mortality independently of adherence to health-enhancing amounts of MVPA is currently debated. While some studies support an independent effect of SB [10,11,15], others have shown that any effects of SB on morbidity and mortality risks are attenuated by engagement in MVPA, roughly approximating the current guideline of ≥150 weekly minutes [12,13]. In terms of metabolic risk in older adults, one previous study concluded that detrimental impacts of high amounts of SB on the presence of MetS were observed only in those spending less than 150 min of MVPA per week, while being mitigated in more physically active individuals [16]. Similar findings support that adherence to the MVPA guideline may offset the adverse associations between time in SB and metabolic risk when expressed as a continuous metabolic risk score rather than a binary MetS outcome [17,18]. Conversely, another study showed that the detrimental association between time in SB and a metabolic syndrome risk score was evident in a group of older adults spending an average of 10-fold the minimum recommended amount of MVPA [19], which questions previous conclusions about detrimental health effects of time in SB to be eliminated by adherence to the MVPA guideline in older adults. Therefore, further investigations are warranted in order to determine how time in SB is linked to clustered metabolic risk, and whether this link is influenced by adherence to the MVPA guideline. Importantly, given that poor dietary quality is regarded as a leading risk factor behind the global disease burden [20], and MetS [21,22], potential confounding effects of dietary habits when examining the role of SB on clustered metabolic risk outcomes need to be considered. Moreover, previous studies have operationalized clustered metabolic risk either as a binary classification (presence/absence of MetS) [23] or as a continuous metabolic syndrome risk score. Given inter-individual differences in number and severity of metabolic risk components, the use of a continuous clustered metabolic risk score may represent a more sensitive approach [24] enabling deeper insights into the role of SB on metabolic risk.

Therefore, the aim of the present study was to investigate how clustered metabolic risk based on a validated continuous metabolic syndrome risk score is associated with objectively assessed time in SB in physically active and inactive older adults, while considering adherence to healthy eating habits.

## 2 Materials and methods

### 2.1 Study design

The present study utilizes baseline data from the NU-AGE study, which was a one-year randomized controlled trial with the aim to determine effects of healthy diet on biomarkers of inflammageing in older men and women recruited from five European study centers (Clermont Ferrand in France, Bologna in Italy, Wageningen in the Netherlands, Warsaw in Poland and Norwich in the United Kingdom). A conceptual framework of the NU-AGE study has been described in detail elsewhere [25,26]. Within the frame of the NU-AGE study, a database covering a comprehensive set of biological, mental, socio-demographic, and lifestyle-related variables was set up to allow future investigations about the complex interactions between determinants of healthy aging. The present study performs cross-sectional analyses on baseline data from the NU-AGE database, including PA behaviors and metabolic risk factors on 1020 older adults (aged 65–79 years). The study follows the Strengthening the Reporting of Observational Studies in Epidemiology (STROBE) reporting guidelines [27].

### 2.2 Participants

A total of 871 older men and women (Italy *n* = 222, Netherlands *n* = 213, Poland *n* = 199, UK *n* = 237) with complete baseline data were included in the present investigation. To be included in the present study, participants had to be aged between 65–79 years, independently living (without nursing assistance) and competent to make own decisions and be free of diagnosed type 2 diabetes. Exclusion criteria included overt disease such as cancer or dementia, organ failure and history of severe heart disease. Written informed consent was obtained from participants, and the study was conducted in accordance with standards set by the Declaration of Helsinki. Ethical approval was obtained from local ethical review boards at each recruiting center (Independent Ethics Committee of the Sant’Orsola ‐ Malpighi Hospital Bologna (Italy‐03/2011/U/Sper), the National Research Ethics Committee East of England (UK‐12/EE/0109), the Wageningen University Medical Ethics Committee (Netherlands‐11/41), and the Bioethics Committee of the Polish National Food and Nutrition Institute (decision of 03.04.2012).

### 2.3 Study procedures

All study procedures were conducted as part of the baseline data collection of the NU-AGE study described above.

#### 2.3.1 Assessment of PA behaviors

Data on PA behaviors were assessed by a hip-mounted accelerometer (Actigraph GT3x, Actigraph, Pensacola, Florida) for one week. All participants were instructed to wear the monitor at all waking hours, with the exception of water activities. Non-wear time was defined as a minimum of 90 min of continuous zero counts [28]. At least 4 days with at least 10 h of wear time per day was required for inclusion and count cut points for time in SB (<100 counts per min), time in light-intensity PA (LPA) (100–2019 counts per min), and MVPA (>2019 counts per min) were applied in accordance with previous work [7,29,30]. Daily accumulated time spent in SB and different intensities of PA were expressed in relation to total monitor wear time (% time). Percent of daily time spent in SB were categorized based on mathematically derived tertiles labelled as low, medium and high amounts of time in SB. Level of adherence to the MVPA guideline of at least 150 min/week were determined as follows: Inactive (<150 min/week) and Active (≥ 150 min/week).

#### 2.3.2 Assessment of anthropometrics and biomarkers of metabolic risk

Body height was recorded to the nearest 0.5 cm using a stadiometer with the participant in a fully erect position standing barefoot with heels together. Body weight was measured to the nearest 0.1 kg using a calibrated scale with the participant wearing light clothes. In subjects standing with arms at the sides and feet positioned closed together, waist circumference (WC) was measured in duplicate at the end of expiration to the nearest 0.1 cm at the midpoint between iliac crest and lower costal margin. Systolic and diastolic blood pressures (SBP and DBP) were determined using automated and calibrated electronic blood pressure monitors (Omron, M2 compact, Milano, Italy. Omron HEM-7117-E, Omron Healthcare, Kyoto, Japan. Omron M2 Basic HEM-7116-E8(v), Omron Healthcare, Kyoto, Japan. Dinamap Pro 100, Welch-Allyn New York, US.). Blood pressures were taken by trained personnel using the following standardized procedure: three readings were recorded with subjects seated with arm supported, feet flat on the floor and with limbs uncrossed. All measurements were obtained on the left arm, following five minutes seated rest as previously described [31]. Mean arterial pressure (MAP) was calculated according to DBP + 1/3(SBP-DBP). Blood samples were collected after an overnight fast by venipuncture from an antecubital vein. Participants were asked to avoid heavy exercise and alcohol in the previous 24-h. Fasting glucose and insulin levels were determined by biochemical assay and chemiluminescent immunoassay, respectively, and the homeostasis model assessment for insulin resistance (HOMA-IR) was calculated according to fasting serum insulin (μU/mL) × fasting plasma glucose (mmol/L)/22.5 [32]. Levels of High-Density Lipoprotein (HDL)-cholesterol and triglycerides were measured on a konelab system (Thermo Electron SA, Cergy-Pontoise, France) using reagents from Thermo Scientific (Asnières sur Seine, France).

#### 2.3.3 Assessment of the continuous metabolic syndrome risk score (cMSy)

The following biomarkers of metabolic risk were used to create a continuous metabolic syndrome risk score (cMSy): WC, HOMA-IR, MAP, and levels of triglycerides and HDL-cholesterol. The cMSy was created based on procedures previously described by Wijndaele et al. [24], where a principal component analysis with varimax rotation was employed to determine weighted component scores explaining major parts of the total variance (eigenvalue > 1.0). In brief, sex-specific analyses were performed on the five normalized metabolic risk variables, which resulted in two components that explained 66% and 65% of the total variance in men and women, respectively. Criteria for generating valid component scores were fulfilled in both sexes (KMO: >0.6; Bartlett’s test of sphericity: p < 0.05). The cMSy was derived in each sex by summing the two individual weighted component scores [24] and merged into one standardized gender-combined variable.

#### 2.3.4 Assessment of covariates

Information on medical history, current use of medication for hypertension and/or blood lipid abnormalities, years of education, marital status (unmarried, married, divorced, widowed), smoking status (never-, former-, current smoker) were self-reported. General health status was assessed based on the 36-item Short Form (SF-36) questionnaire. Adherence to healthy eating habits was determined based on a healthy dietary index score derived from seven-day food records. Participants received written instructions and had face-to-face training about how to describe foods and amounts consumed using preformatted food records, including eight meal occasions. Dieticians were trained across study centers in reviewing the completed food records and all records were subsequently coded and translated into nutrients exploiting the local food composition tables in each recruiting center (NEVO 2011 in The Netherlands, McCance and Widdowson’s in The UK, BDA and INRAN in Italy, NFNI in Poland) and softwares (NetWISP v4.0, Tinuviel Software, Warrington, UK; Compl-eat, Wageningen university & research, Wageningen, The Netherlands; DIETA-5, National Food and Nutrition Institute, Warsaw, Poland). The NU-AGE healthy diet score has been described in detail elsewhere [33]. In brief, the NU-AGE healthy diet score is based on adherence to dietary guidelines for older adults in the countries included in the NU-AGE study. The healthy diet score is based on 16 food items, where participants are assigned a score ranging from 0 to 10 according to the level of adherence to recommended amounts for each food item. In total, the healthy diet score ranges from 0 to 160 points, where 160 points represent the highest adherence. Consumption above minimum recommended amounts to the following beneficial food items yielded 10 points: fruits, vegetables, legumes, low-fat dairy, low-fat cheese, fish, low-fat meat and poultry, nuts, olive oil, fluids, and vitamin D (supplement). In contrast, for moderation food items (alcohol, salt, and sweets), intakes below a maximum amount received 10 points [33].

### 2.4 Statistical analysis

Data on participant characteristics are presented as means ± standard deviation (SD), median and interquartile range (IQR) and percentages. Skewed data were transformed to fit a normal distribution. Effects on standardized cMSy across SB tertiles (High, Medium, Low) in groups with and without adherence to the MVPA guideline (Active/Inactive) were analyzed by general linear modelling (analysis of covariance; ANCOVA). All analyses were adjusted by study center, sex, marital status, smoking status, use of prescribed medication for hypertension and/or blood lipid abnormalities as fixed factors, and by age, education years, general health status and the NU-AGE healthy diet score as continuous covariates. As any effect on cMSy by level of SB tertile may be confounded by differences in total MVPA time within active and inactive groups, total accumulated time in MVPA (expressed as % of total daily time) was added as a continuous covariate when analyzing effects on cMSy across SB tertiles. Post-hoc comparisons between SB tertiles within active and inactive groups were conducted with Bonferroni correction for multiple comparisons. Effects on cMSy between active and inactive groups were further re-analyzed within each SB tertile, with adjustment for potential differences in daily accumulated SB time (expressed as % of total daily time). Outcomes from ANCOVA are presented as mean ± SEM. Assumptions for general linear modelling were checked, including normality and homogeneity of variances. A priori power calculation revealed that small to moderate effect sizes (≥ 0.25) would be detected using the ANCOVA model with a power of ≥80% and with alpha (α) set to *p* < 0.05. All analyses were performed using SPSS version 28 (Armonk, NY, USA: IBM Corporation).

## 3 Results

A total of 871 older European adults (363 men and 508 women, mean age: 71 ± 4 years) with complete data were included in the analyses. Participant characteristics across mathematically derived tertiles of daily time in SB are shown in Table 1. The majority of participants were married, with an average of approximately 13 years of education and did not use prescribed medication for hypertension and/or blood lipid abnormalities. Nearly one half of the population were former or current smokers, and the general health status was similar across SB tertiles. Distribution of time spent in different intensities of PA across tertiles of SB is shown in Table 1. PA behaviors were assessed during 7.0 ± 0.6 days, with an average wear time of 14.6 ± 0.9 h per day. On an average day, the study population spent 60%, 37% and 3% of the total monitor wear time in SB, in LPA, and in MVPA, respectively. On average, time in SB differed by approximately 2.5 h mirrored by a similar difference in LPA time between the low and high SB tertiles. Further, participants in the low SB tertile had about twice the amount of time in MVPA compared to the high SB tertile. Half of the study population accumulated at least 150 weekly minutes of MVPA and were classified as physically active, with the largest proportion among the low SB group (Table 1). A description of metabolic risk components included in the calculation of the cMSy across tertiles of SB is presented in Table 2.

**Table 1:** Participant characteristics across tertiles of daily time in sedentary behaviors (SB).

	Low SB (<8.3 h/day)	Medium SB (8.3–9.3 h/day)	High SB (>9.3 h/day)
No. of participants	290	291	290
Age (years)	70.0 ± 3.7	71.28 ± 4.0	71.5 ± 4.0
Sex (%)			
Female	65.2	59.8	50.0
Male	34.8	40.2	50.0
Study center (%)			
Italy	29.7	25.1	21.7
UK	23.8	28.5	29.3
The Netherlands	19.0	23.7	30.7
Poland	27.5	22.7	18.3
Marital status (%)			
Unmarried	2.4	3.4	3.4
Married	68.3	63.9	70.3
Divorced	13.1	12.8	10.4
Widowed	16.2	19.9	15.9
Smoking status (%)			
Never	54.8	52.2	50.3
Former	39.7	43.3	46.6
Current	5.5	4.5	3.1
Education (years)	12.8 ± 3.7	12.7 ± 3.6	12.7 ± 3.5
Medication use (%)			
Yes	23.1	30.6	29.7
No	76.9	69.4	70.3
General health score (0−100)	66.7 ± 16.5	68.5 ± 15.0	67.9 ± 17.4
NU-AGE diet score (0−100)	50.4 ± 9.6	50.3 ± 9.1	49.3 ± 9.4
Daily time in PA behaviors			
Total wear time (h/day)	14.6 ± 0.9	14.6 ± 0.9	14.5 ± 0.9
Time in SB (h/day)	7.5 ± 0.8	8.8 ± 0.6	10 ± 0.9
Time in LPA (h/day)	6.5 ± 0.8	5.3 ± 0.6	4.2 ± 0.7
Time in MVPA (min/day)	36 ± 25	27 ± 19	19 ± 14
Physically active (%)			
Yes	66.2	55.0	35.9
No	33.8	45.0	64.1

Data show mean value ± standard deviation unless noted.

Abbreviations: PA, physical activity; SB, sedentary behaviors, LPA, light-intensity physical activity; MVPA, moderate-to-vigorous physical activity.

**Table 2:** Metabolic risk components included in the continuous metabolic syndrome risk score (cMSy) across tertiles of sedentary behaviors (SB).

	Low SB (<8.3 h/day)	Medium SB (8.3–9.3 h/day)	High SB (>9.3 h/day)
Waist circumference (cm)	88.6 ± 10.9	92.0 ± 10.7	95.5 ± 12.3
SBP (mmHg)	136 ± 17.9	141 ± 21.3	141 ± 19.7
DBP (mmHg)	73.5 ± 9.9	75.5 ± 11.3	76.2 ± 10.1
Triglycerides (mg/dL)	95.2 ± 36.0	109.2 ± 44.9	112.3 ± 50.7
HDL-cholesterol (mg/dL)	64.7 ± 18.0	58.7 ± 17.0	55.2 ± 17.2
Glucose (mg/dL)	97.7 ± 11.6	98.6 ± 10.9	100.2 ± 13.6
Insulin (μU/L)^a^	7.0 (4–11)	8.0 (5–12)	8.0 (5–12)
HOMA-IR^a^	1.73 (1.02–2.74)	1.83 (1.17–2.91)	1.99 (1.22–3.10)

Data show mean value ± standard deviation unless noted.

Abbreviations: SBP, systolic blood pressure; DBP, diastolic blood pressure; HDL-cholesterol, High-Density Lipoprotein cholesterol; HOMA-IR, Homeostatic Model Assessment of Insulin Resistance.

a Expressed as median and interquartile range.

**Fig. 1:**
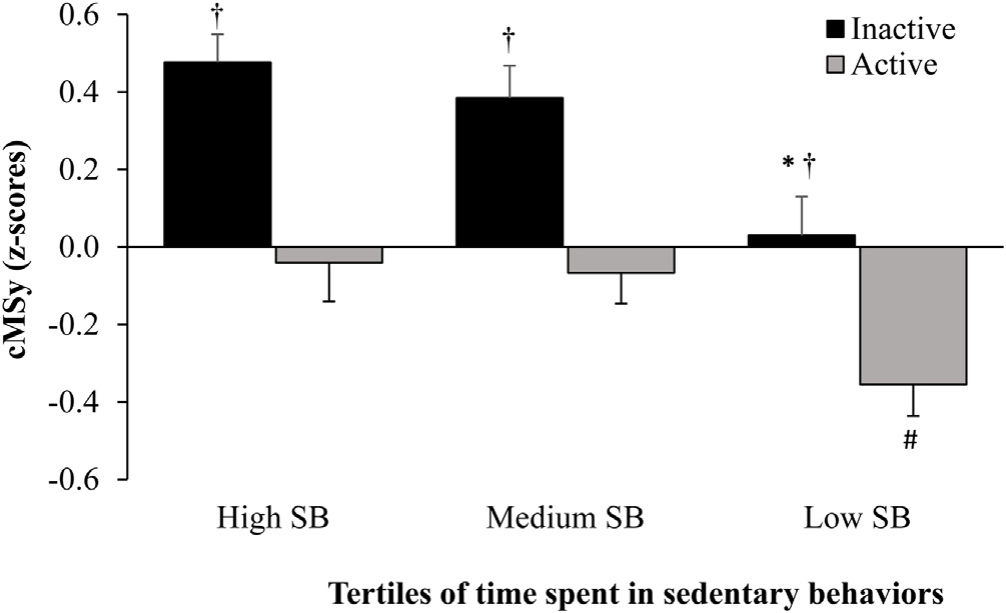
Continuous metabolic syndrome risk score (cMSy) across tertiles (High, Medium, Low) of sedentary behaviors (SB) stratified by adherence to the MVPA guideline (Active, Inactive). Data are means ± SEM adjusted by sex, age, study center, marital status, years of education, general health status, smoking, current use of medication for hypertension and/or blood lipid abnormalities, adherence to the NU-AGE healthy diet score, and total time in MVPA and SB in comparisons across SB tertiles and active/inactive groups, respectively. *Significant difference (*p* < 0.05) compared to higher SB tertiles within inactive groups. #Significant difference (*p* < 0.05) compared to higher SB tertiles within active groups. †Significant difference (*p* < 0.05) between active and inactive groups within each tertile of SB time.

Stratification of SB tertiles by level of adherence to the MVPA guideline resulted in six mutually exclusive PA behavior groups according to the following: High SB-Inactive (*n* = 186), High SB-Active (*n* = 104), Medium SB-Inactive (*n* = 131), Medium SB-Active (*n* = 160), Low SB-Inactive (*n* = 98), Low SB-Active (*n* = 192). Results from the analysis of cMSy by tertiles of SB (High, Medium, Low) in active and inactive older adults are presented in Fig. 1. Significant main effects on the cMSy were observed across tertiles of SB in both inactive (F (2, 399) = 9.145, *p* < 0.05) and active (F (2, 440) = 6.799, *p* < 0.05) groups after adjustment by level of adherence to healthy eating habits and daily accumulated time in MVPA, with a trend of lower cMSy by lower tertiles of SB time (Fig. 1). Post hoc comparisons revealed that older adults in the low SB group (1st tertile) had a significantly lower cMSy compared to those in the high SB group (3nd tertile) in both inactive (mean cMSy difference: −0.49, 95% confidence interval (CI): −0.77, −0.21) and active (mean cMSy difference: −0.36, 95% CI: −0.65, −0.07) older adults. The beneficial impact of belonging to the low SB group remained evident when comparing average cMSy with those belonging to the medium SB group (2rd tertile) in inactive (mean cMSy difference: −0.39, 95% CI: −0.68, −0.09) as well as active (mean cMSy difference: −0.33, 95% CI: −0.58, −0.09) older adults (Fig. 1). In contrast, no significant differences in cMSy were observed between the high and medium SB tertiles (2nd vs. 3rd) in either active or inactive groups.

We further examined the differences in cMSy between active and inactive groups within each tertile of SB time, including adjustment for differences in total accumulated time in SB (Fig. 1). Results showed that the active compared to the inactive groups had a significantly lower cMSy across all SB tertiles, indicating that adherence to the MVPA guideline is related to beneficial effects on metabolic health even in older adults accumulating higher volumes of daily SB. Mean differences in cMSy between active and inactive groups corresponded to −0.29 (95% CI: −0.53, −0.06), −0.27 (95% CI: −0.51, −0.03) and −0.37 (95% CI: −0.60, −0.14) in the low, medium and high SED tertiles, respectively (Fig. 1).

## 4 Discussion

A novel finding from the present study was that low daily time in SB is related to lower clustered metabolic syndrome risk in both physically active and inactive older adults and that this beneficial link is evident independently of healthy eating habits. A PA behavior characterized by low amounts of daily time in SB combined with MVPA adherence was related to the lowest metabolic risk, reinforcing the importance of targeting low amounts of SB alongside MVPA adherence for promotion of metabolic health in older adults.

A recent study reported detrimental associations between time in SB and clustered metabolic risk, where older adults with high SB and low MVPA had a high metabolic risk score [17]. Nevertheless, whether differences in time in SB among physically active groups may impact on metabolic risk remained unanswered. Here we show that those belonging to the lowest SB tertile had a lower metabolic syndrome risk compared to their peers accumulating more time in SB, and that this beneficial impact was evident regardless of being physically active or not. Importantly, as the total accumulated time in MVPA may differ between individuals adhering to the MVPA guideline (≥ 150 min/week), we further adjusted our analysis by time in MVPA to rule out potential confounding effects on metabolic syndrome risk by engagement in high amounts of MVPA (e.g., ≥ 300 min/week). Therefore, our findings lend support to the role of SB as an independent risk factor for metabolic risk in older adults [15,19]. Interestingly, no differences in metabolic syndrome risk were observed between medium and high SB tertiles. This suggests a non-linear association between SB and metabolic health, where lower metabolic syndrome risk scores were evident only below a threshold of 8.3 h/day, which represented the cut-point between the low and medium tertile in our data set. Similarly, results from a previous meta-analysis reported a threshold of 8.5 h/day of SB from which all-cause mortality risk increases in inactive groups [13]. Furthermore, although not based on older adults, a compilation of studies showed that increases in type 2 diabetes biomarkers occur above a threshold corresponding to 7−9 h/day of SB [34] which covers the SB threshold of 8.3 h/day evidenced in our study in older adults.

Alongside the observed beneficial effects of limited amounts of time in SB on metabolic syndrome risk, older adults accumulating ≥ 150 weekly min of MVPA had consistently lower metabolic syndrome risk compared to their inactive peers within each tertile of SB. This is in line with previous studies revealing that increased time in MVPA is related to lower metabolic risk scores [18] and lower likelihood of having MetS in older adults [35], suggesting that promotion of metabolic health in older adults may be optimized by combining MVPA adherence with low amounts of time in SB.

Interestingly, previous studies have indicated that time in LPA (i.e. below the moderate-intensity threshold) can also contribute to better metabolic health [36,37]. In the inactive group, having a low amount of SB was linked to better metabolic health despite low amounts of MVPA. Therefore, as less time in SB is mainly translated into more time in LPA, our data suggests that engagement in physical activities below the moderate intensity threshold can contribute to better metabolic health, which is encouraging for older adults who may find adherence to the MVPA guideline challenging.

### 4.1 Strengths and limitations

To the best of our knowledge, this is the first study that investigates associations between continuous metabolic syndrome risk and combinations of objectively assessed PA behaviors in older adults, while considering adherence to healthy eating habits. Although the quality of dietary intake is a well-established risk factor for chronic diseases [20], studies examining associations between PA behaviors and metabolic risk in older adults have rarely accounted for healthy eating habits. This is unfortunate as a previous investigation confirmed that adherence to the MVPA guideline is associated with higher adherence to healthy eating habits [38]. Hence, the ability to separate influences of PA behaviors from those of diet has been limited in most previous investigations. Therefore, inclusion of adherence to healthy eating habits in our work represents a novel strength. Another strength includes the use of a continuous metabolic syndrome risk score based on established metabolic risk components, including a strong proxy for insulin resistance (HOMA-IR). The integration of individual metabolic risk factors into a continuous metabolic syndrome risk score is regarded as a more sensitive tool to reflect metabolic risk compared to the binary definition of MetS [24]. Other strengths of the present study include the objectively assessed time spent in PA behaviors, the relatively large sample of older men and women covering four different European countries, as well as the use of a comprehensive set of covariates, including sociodemographic, medical and lifestyle-related factors. However, the study is not without limitations. Our findings are based on a cross-sectional analysis that limits conclusions about causality. Given that our sample excluded older adults being frail, having dementia or with a history of severe heart disease, care should be taken when generalizing the study findings to wider populations of older adults. Of note, an average of 60% of the monitor wear time (corresponding to 8.8 h in our data) was spent in SB, which is in line with previous data on national samples of older Swedish and US adults [7,8]. Although accelerometers represent a valid tool for assessment of PA behaviors, including SB [39], it may be less accurate in reflecting actual energy costs for PA involving heavy lifting, stair climbing or bicycling. Moreover, even though some non-movement activities (e.g. standing) may be incorrectly classified as SB, more than 80% of sedentary activities in a sitting or lying position have been shown to be correctly identified [40].

### 4.2 Future directions

While our findings indicated the existence of a non-linear dose-response relationship between time in SB and cMSy, future studies are needed to further elucidate the shape of such proposed relationship and take advantage of experimental designs in order to evaluate effects of different amounts of time in SB on metabolic syndrome risk in older populations. Furthermore, this should be addressed in various subgroups of the older population given the large variations in general health status, which may moderate relationships between SB and metabolic health. Furthermore, future investigations should specifically explore the benefits of time in LPA on metabolic health in older populations to determine health-enhancing effects of PA at intensities below the current recommendation.

## 5 Conclusions

Low amounts of daily time spent in SB are related to lower metabolic syndrome risk in both physically active and inactive older adults, and regardless of adherence to healthy eating habits. The study findings reinforce the importance of targeting low amounts of SB alongside adherence to the MVPA guideline for promotion of metabolic health in older adults.

## Funding

This study was supported by grants from the European Union’s Seventh Framework Program, no. 266486 (NU-AGE: New dietary strategies addressing the specific needs of the elderly population for healthy aging in Europe); the Kamprad family foundation for entrepreneurship, research & charity, no. 202110070; the Swedish Heart-Lung foundation, no. 20240877.

## Declaration of competing interest

The authors declare that they have no known competing financial interests or personal relationships that could have appeared to influence the work reported in this paper.
